# Supplementary material for: Strand Displacement Chain Reaction (SDCR): New Hybrid Amplification Technique for Fast and Sensitive Detection of Genetic Materials
Source: Biomolecules. 2025 Sep 12;15(9):1313. doi: 10.3390/biom15091313 (PMC12467831; doi:10.3390/biom15091313)
Supplement: Supplementary file 1 [file biomolecules-15-01313-s001.zip › Figure S1.pdf]

Supplementary Materials: Figure S1.

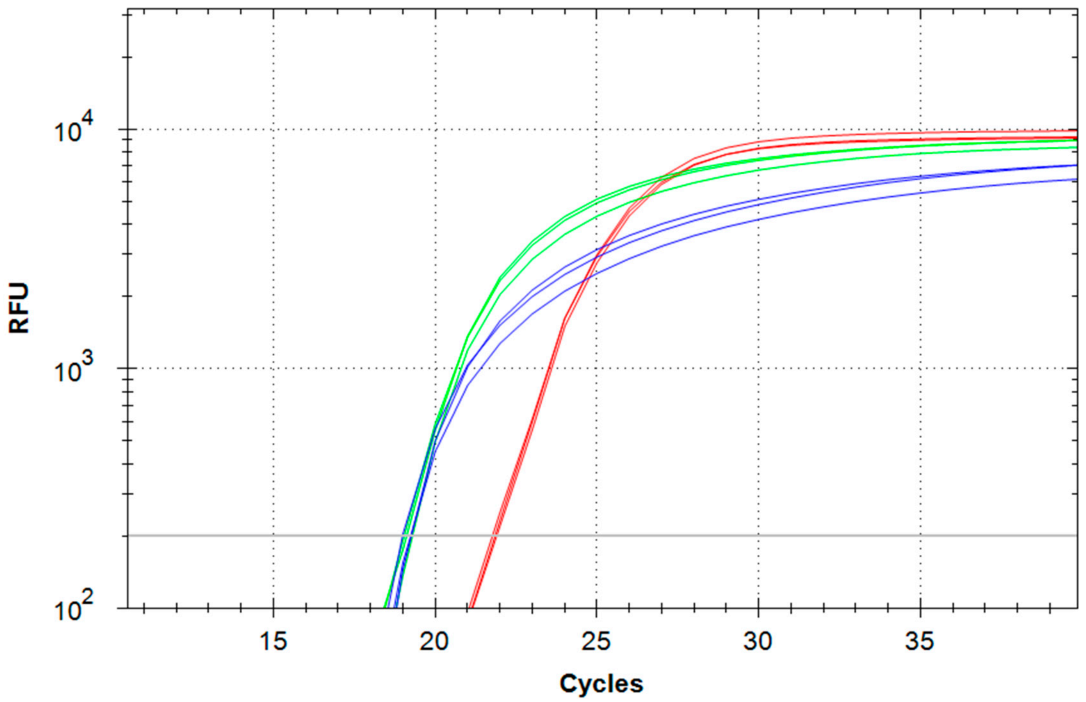

Figure S1. Optimization of SD polymerase concentrations for qSDCR assays.

qSDCR assays of human beta-2-microglobulin (B2M) cDNA contained 0.5 pg of the human total cDNA library per reaction and different concentrations of SD DNA polymerase: 0.4 U/µL (red curves); 0.8 U/µL (green curves); 2 U/µL (blue curves). The reactions were carried out in three replicates. Concentrations over 0.8 U/µL did not improve the performance of qSDCR.
