# Supplementary material for: Strand Displacement Chain Reaction (SDCR): New Hybrid Amplification Technique for Fast and Sensitive Detection of Genetic Materials
Source: Biomolecules. 2025 Sep 12;15(9):1313. doi: 10.3390/biom15091313 (PMC12467831; doi:10.3390/biom15091313)
Supplement: Supplementary file 1 [file biomolecules-15-01313-s001.zip › Figure S2.pdf]

Methods Slopes Comparison

Introduction

All calculations were conducted using R language. For linear models slopes test we used **ANOVA**. If *p* values are **lower than 0.05** we stated that difference is statistically meaningful.

Loading Cq data

All Cqs are stored in one csv file which has such columns:

- Copies – input amount
- Cq – obtained Cq after PCR
- Method – PCR method (*qSDCR*, *qPCR_1*, *qPCR_2*)
- Kit – PCR target (*Lambdavirus*, *Mycobacterium*, *B2M*)

After loading data we adding log10_copies column (log10 of input) to make fits linear.

```
library(tidyverse)
```

```
── Attaching core tidyverse packages ──────────────────────── tidyverse 2.0.0 ──
✔ dplyr     1.1.4     ✔ readr     2.1.5
✔ forcats   1.0.0     ✔ stringr   1.5.1
✔ ggplot2   3.5.2     ✔ tibble    3.3.0
✔ lubridate 1.9.4     ✔ tidyr     1.3.1
✔ purrr     1.1.0
── Conflicts ────────────────────────────────────────── tidyverse_conflicts() ──
✖ dplyr::filter() masks stats::filter()
✖ dplyr::lag()    masks stats::lag()
ℹ Use the conflicted package (<http://conflicted.r-lib.org/>) to force all conflicts to become errors
```

```
library(lsmeans)
```

```
Loading required package: emmeans
Welcome to emmeans.
Caution: You lose important information if you filter this package's results.
See '? untidy'
The 'lsmeans' package is now basically a front end for 'emmeans'.
Users are encouraged to switch the rest of the way.
See help('transition') for more information, including how to
convert old 'lsmeans' objects and scripts to work with 'emmeans'.
```

```
library(ggpmisc)

Loading required package: ggpp
Registered S3 methods overwritten by 'ggpp':
  method                  from
  heightDetails.titleGrob ggplot2
  widthDetails.titleGrob  ggplot2

Attaching package: 'ggpp'

The following object is masked from 'package:ggplot2':

    annotate

cqData <- read.csv("cq_data.csv") |>
  mutate(log10_copies = log10(Copies))
```

Slopes comparison

Lambdavirus (fig. 2 in main article)

```
dat <- cqData |> filter(Kit == "Lambdavirus")
fit <- lm(Cq ~  log10_copies * Method, data = dat)
anova(fit)

Analysis of Variance Table

Response: Cq
                    Df Sum Sq Mean Sq F value    Pr(>F)
log10_copies         1 318.18  318.18  2649.0 < 2.2e-16 ***
Method               2 771.77  385.88  3212.6 < 2.2e-16 ***
log10_copies:Method  2  28.90   14.45   120.3 9.105e-15 ***
Residuals           29   3.48    0.12
---
Signif. codes:  0 '***' 0.001 '**' 0.01 '*' 0.05 '.' 0.1 ' ' 1

trends <- lstrends(fit, "Method", var="log10_copies")
trends

 Method log10_copies.trend     SE df lower.CL upper.CL
 qPCR_1              -3.04 0.0895 29    -3.22    -2.85
 qPCR_2              -3.95 0.0978 29    -4.15    -3.75
 qSDCR               -1.90 0.0895 29    -2.09    -1.72

Confidence level used: 0.95

pairs(trends)

 contrast        estimate    SE df t.ratio p.value
 qPCR_1 - qPCR_2     0.91 0.133 29   6.859  <.0001
 qPCR_1 - qSDCR     -1.13 0.127 29  -8.953  <.0001
```

```
 qPCR_2 - qSDCR     -2.04 0.133 29 -15.404  <.0001

P value adjustment: tukey method for comparing a family of 3 estimates
```

```
ggplot(dat, aes(x= log10_copies, y = Cq, color = Method)) +
  geom_point() +
  stat_poly_line() +
  stat_poly_eq(use_label(c("eq", "R2"))) +
  theme_bw()
```

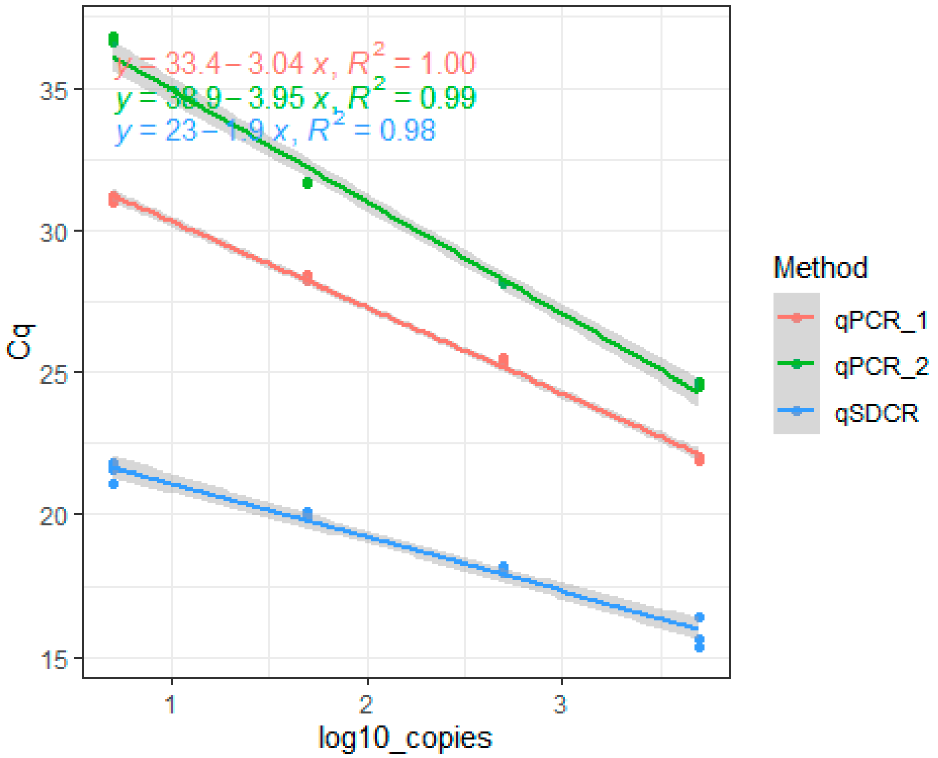

From fits pairs comparison we can see that all methods slopes (trend in *lstrends* function output terms) is markedly differs from each other.

Mycobacterium (fig. 3 in main article)

```
dat <- cqData |> filter(Kit == "Mycobacterium")
fit <- lm(Cq ~  log10_copies * Method, data = dat)
anova(fit)

Analysis of Variance Table

Response: Cq
                    Df  Sum Sq Mean Sq  F value    Pr(>F)    
log10_copies         1  405.06  405.06 1771.057 < 2.2e-16 ***
Method               2 1610.58  805.29 3521.004 < 2.2e-16 ***
log10_copies:Method  2   23.43   11.71   51.221  2.12e-10 ***
Residuals           30    6.86    0.23
```

```
---
Signif. codes:  0 '***' 0.001 '**' 0.01 '*' 0.05 '.' 0.1 ' ' 1

trends <- lstrends(fit, "Method", var="log10_copies")
trends

 Method log10_copies.trend    SE df lower.CL upper.CL
 qPCR_1                -3.46 0.123 30    -3.71    -3.20
 qPCR_2                -3.56 0.123 30    -3.82    -3.31
 qSDCR                 -1.98 0.123 30    -2.23    -1.73

Confidence level used: 0.95

pairs(trends)

 contrast        estimate    SE df t.ratio p.value
 qPCR_1 - qPCR_2    0.108 0.175 30   0.617  0.8122
 qPCR_1 - qSDCR    -1.474 0.175 30  -8.441  <.0001
 qPCR_2 - qSDCR    -1.582 0.175 30  -9.057  <.0001

P value adjustment: tukey method for comparing a family of 3 estimates

ggplot(dat, aes(x= log10_copies, y = Cq, color = Method)) +
  geom_point() +
  stat_poly_line() +
  stat_poly_eq(use_label(c("eq", "R2"))) +
  theme_bw()
```

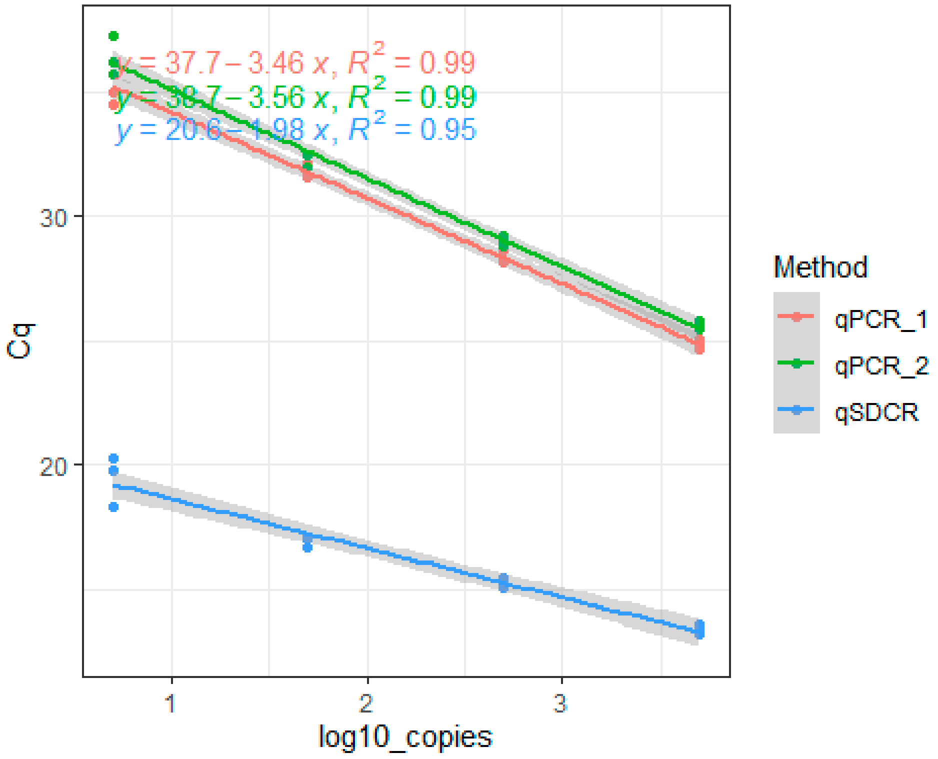

From fits pairs comparison we can see that qSDCR method slope (trend in *lstrends* function output terms) is markedly differs from qPCR_1 and qPCR_2 methods slopes. But qPCR_1 and qPCR_2 methods slopes don't differ from each other.

B2M (fig. 4)

```
dat <- cqData |> filter(Kit == "B2M")
fit <- lm(Cq ~  log10_copies * Method, data = dat)
anova(fit)

Analysis of Variance Table

Response: Cq
                    Df Sum Sq Mean Sq F value    Pr(>F)
log10_copies         1 378.16  378.16 9083.25 < 2.2e-16 ***
Method               2 704.01  352.01 8455.03 < 2.2e-16 ***
log10_copies:Method  2  21.49   10.74  258.04 < 2.2e-16 ***
Residuals           30   1.25    0.04
---
Signif. codes:  0 '***' 0.001 '**' 0.01 '*' 0.05 '.' 0.1 ' ' 1

trends <- lstrends(fit, "Method", var="log10_copies")
trends

 Method log10_copies.trend     SE df lower.CL upper.CL
 qPCR_1                -3.47 0.0527 30    -3.58    -3.36
 qPCR_2                -3.30 0.0527 30    -3.41    -3.19
 qSDCR                 -1.93 0.0527 30    -2.03    -1.82

Confidence level used: 0.95

pairs(trends)

 contrast        estimate     SE df t.ratio p.value
 qPCR_1 - qPCR_2   -0.171 0.0745 30  -2.291  0.0726
 qPCR_1 - qSDCR    -1.544 0.0745 30 -20.719  <.0001
 qPCR_2 - qSDCR    -1.373 0.0745 30 -18.428  <.0001

P value adjustment: tukey method for comparing a family of 3 estimates

ggplot(dat, aes(x= log10_copies, y = Cq, color = Method)) +
  geom_point() +
  stat_poly_line() +
  stat_poly_eq(use_label(c("eq", "R2"))) +
  theme_bw()
```

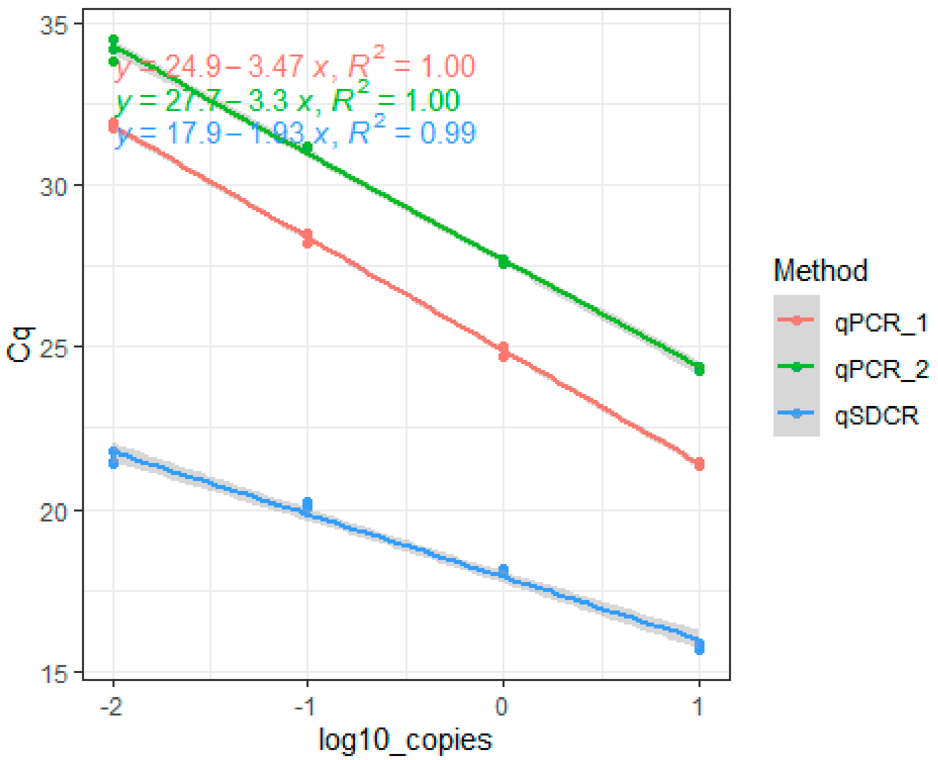

From fits pairs comparison we can see that qSDCR method slope (trend in *lstrends* function output terms) is markedly differs from qPCR_1 and qPCR_2 methods slopes. But qPCR_1 and qPCR_2 methods slopes don't differ from each other.
